# Sorption Studies of Tetracycline Antibiotics on Hydroxyapatite (001) Surface—A First-Principles Insight

**DOI:** 10.3390/ma15030797

**Published:** 2022-01-21

**Authors:** Jiaming Song, Naiyu Cui, Xuran Mao, Qixuan Huang, Eui-Seok Lee, Hengbo Jiang

**Affiliations:** 1 The Conversationalist Club, School of Stomatology, Shandong First Medical University, Shandong Academy of Medical Sciences, Tai’an 271016, China; jiamingsongpp@outlook.com (J.S.); Cuinaiyuuu@outlook.com (N.C.); mxr021227@outlook.com (X.M.); xianliang_h@163.com (Q.H.); 2 Department of Oral and Maxillofacial Surgery, Graduate School of Clinical Dentistry, Korea University, Seoul 08308, Korea

**Keywords:** bone targeting, tetracycline, hydroxyapatite, drug delivery, density functional theory

## Abstract

Owing to the limitations of traditional systemic drug delivery in the treatment of bone diseases with side effects on normal cells, the selection of materials with high affinities for bones, as targeting ligands to modify drug carriers, has become an important research topic. Tetracyclines (TCs) have an adsorption effect on hydroxyapatite (HAp). Thus, they can be used as bone-targeting ligands and combined with drug carriers. In this study, density functional theory is used to analyze the interaction mechanism of TC, oxytetracycline (OTC), chlortetracycline, and HAp. We calculate the electrostatic potential (ESP) and molecular orbitals to predict the possible binding sites of TCs on the HAp surface. The adsorption energy is used to compare the affinities of the three TCs to HAp. An independent gradient model analysis is performed to study the weak interaction between TCs and HAp. The coordination bond between TCs and the HAp surface is evaluated by conducting a charge density difference analysis. The results show that OTC has the highest affinity to HAp because the introduction of hydroxyl groups change the adsorption configuration of OTC. Thus, OTC adsorbed on HAp in a broken-line shape exposes more binding sites. This study provides a theoretical basis for TCs as bone-targeting ligands in treating bone diseases and in improving the safety of treatment by selecting different bone-targeting ligands.

## 1. Introduction

Osteoporosis, bone metastases, and other bone-related diseases are major health problems [1,2]. They are often treated using traditional systemic drug delivery methods, such as oral and intravenous injections. However, these methods have disadvantages such as a low local drug concentration and poor absorption of the drug by bone tissue [3,4]. In addition, the drug may be excreted before reaching the delivery target; thus, a large dose of the drug increases medical costs and causes potential systemic side effects [5]. A more ideal method for the treatment of bone diseases is to concentrate therapeutic drugs in the bone tissue to maximize the local therapeutic effect of the drugs [5,6]. Bone-targeting can be employed to accomplish this type of local administration. Bone-targeting [7] selects materials with high affinities to bones as targeting ligands to modify drug carriers for delivering drugs to their destinations. The drug can be delivered to the expected target, thus reducing not only the side effects on normal cells but also the dosage and frequency of medication [8,9]. Therefore, bone-specific drug delivery systems for local drug delivery have gained considerable research interest [10,11,12,13].

Hydroxyapatite (HAp) is the main inorganic mineral component of bones [8,14,15]. The molecular formula of HAp is Ca_10_(PO_4_)_6_(OH)_2_, which consists of very small hexagonal crystalline proteins, usually no more than 200 Å in size. An HAp cell consists of a hexagonal crystal structure with lattice parameters a = 0.951 nm, C = 0.684 nm, and space group *P*6_3_/*m* [16]. Bystrov et al. described the results of interactions between HAp nanostructures and a variety of substances, including carbon nanotubes (CNTs), citrate, and living cell-osteoblasts, using first-principles methods and quantum chemical calculations including DFT [17]. Owing to bone diseases, HAp can be exposed to the blood [18], making it a potential target for the delivery of diagnostic agents to the bone [19]. As a new type of drug delivery system, nanodrug delivery systems have garnered significant research interest for bone therapy [20]. Nanoparticles (NPs) enable drugs to be released in a controlled and sustainable manner [21,22]. In addition, the surface of an NP can be coupled with a targeting ligand to target the intended bone tissue. Tetracyclines (TCs) are bone-specific targeting ligands [19].

TCs are a class of typical broad-spectrum antibiotics [23]. TC, oxytetracycline (OTC), and chlortetracycline (CTC) are commonly used in medicine [24]. They exhibit various advantages including a high oral bioavailability, low price, and accessibility [13,25,26,27,28]. TCs have a high affinity to HAp. Fluorescent labeling shows that the surface of a mineralized dead bone absorbs TCs. It is speculated that TC is combined with HAp in the bone [29]. In addition, TC, OTC, and CTC can adsorb HAp [30,31,32,33]. Therefore, TCs are promising targeting ligands.

TC-based bone-targeted drug delivery systems have been studied. Neale et al. combined TCs and estradiol to synthesize bone-targeted estrogen to treat osteoporosis and to improve the safety of osteoporosis treatment [13]. Wang et al. used the esterification reaction of poly(lactic-co-glycolic acid) (PLGA) and TCs to develop NPs carrying simvastatin (SIM) to target bone tissue to treat osteoporosis [11]. Xie et al. studied TC-polyethylene glycol-PLGA micelles loaded with atorvastatin to target osteoporosis [10]. A subsequent study on the SIM carrier showed that bone density, bone strength, and bone mineral content were improved [12].

TCs have a low affinity to bone sites, characterized by a low bone turnover [34]. Numerous functional groups exist on the surface of TCs and are unstable during chemical modifications [35], limiting the development of TCs as a drug delivery system for bone-targeting ligands. Although the effective adsorption capacity of TCs on HAp has been extensively studied in experiments [29,30,31,32,33,36,37], understanding of the specific interaction mechanism between HAp and TCs is limited. The adsorption process of TCs (TC, OTC, and CTC) with different properties on HAp must be further studied.

In this study, density functional theory (DFT) is used to analyze the interaction mechanism of three TCs (TC, OTC, and CTC) and HAp. The electrostatic potential (ESP), molecular orbital, and adsorption energy of the three types of TCs and HAp are calculated. An independent gradient model (IGM) is created and charge density difference analyses are conducted to study the adsorption between TCs and HAp.

## 2. Theoretical Methods

### 2.1. Model Development

**Scheme 1:**
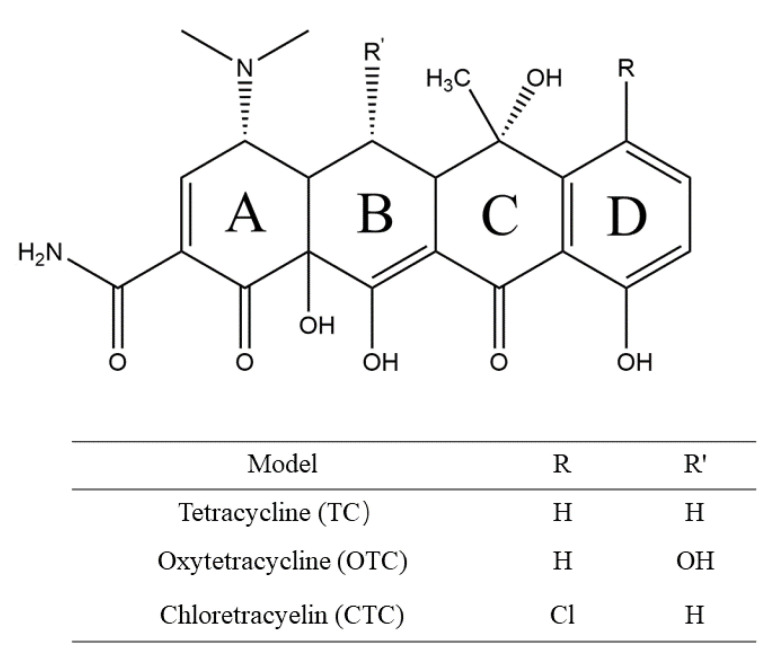
Chemical structures of TC, OTC, and CTC.

The structures of the three TCs (TC, OTC, and CTC) contain the naphthacene skeleton, as shown in Scheme 1. The (001) crystal plane of HAp was selected, and 2 × 2 × 1 supercells were used as the adsorption surfaces of the TCs. A 40 Å vacuum plate was constructed to avoid interactions between adjacent cells.

### 2.2. DFT Calculation

CP2K was used for the geometric structure optimization [38], which was performed using the Broyden–Fletcher–Goldfarb–Shanno algorithm. The Perdew–Burke–Ernzerhof functional [39] was used for the DFT calculations. We used a plane-wave cutoff of 500 Ry and the MOLOPT pseudopotential basis set, which was a completely generalized contraction basis set. We added DFT-D3 (BJ) dispersion correction to more accurately describe weak interactions [40].

## 3. Results and Discussion

### 3.1. ESP

The ESP is a real space function that is most closely related to the electrostatic effect [41,42], which can be used to analyze various electrostatic-dominated weak interactions. When molecules interact with each other, the positive and negative ESPs attract each other. The distribution of the molecular ESP can be analyzed to predict and explain the mode and mechanism of interaction between molecules.

**Figure 1:**
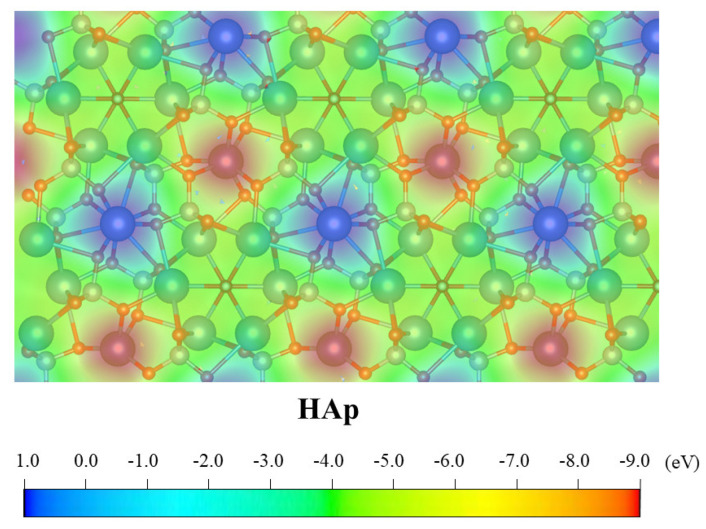
ESP of HAp. The blue area represents a positive ESP exhibiting electrophilic properties, whereas the red area represents a negative ESP exhibiting nucleophilic properties.

The ESP of HAp is shown in Figure 1. A positive ESP is gathered near the Ca atoms in HAp; thus, the Ca atoms are electrophilic. During adsorption, Ca atoms easily interact with atoms with negative ESPs in TCs. Most of the negative ESPs are concentrated near the phosphate groups in HAp. During the adsorption process, the atoms or groups with positive ESPs in the TCs interact with the phosphate groups in HAp.

**Figure 2:**
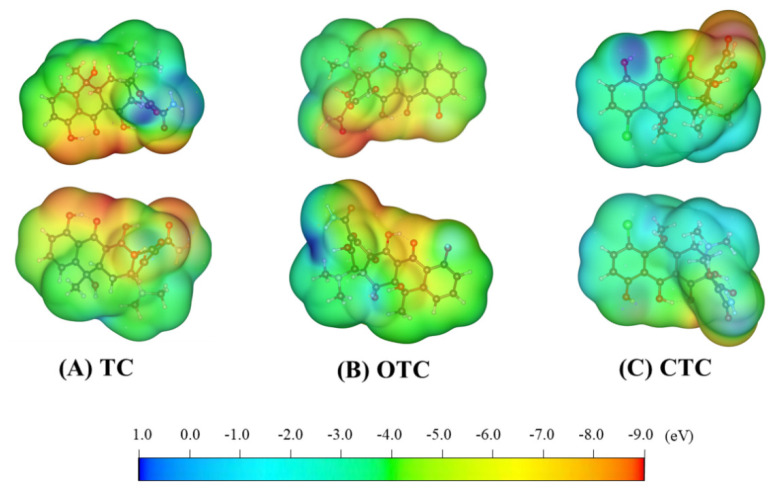
ESPs of (**A**) TC, (**B**) OTC, and (**C**) CTC molecules (two-side view). The blue area represents a positive ESP exhibiting electrophilic properties, whereas the red area represents a negative ESP exhibiting nucleophilic properties.

The ESP of the TC molecules is shown in Figure 2A. All hydrogen atoms have a positive ESP. The hydrogen atoms on the A ring are represented in darker blue, indicating that they have a high positive ESP. This is an active site; thus, it may easily form a hydrogen bond with the phosphate group in HAp during the adsorption process. The surface of the oxygen atoms on the TC has a high negative ESP; thus, it can form a coordination bond with the Ca atoms in HAp.

The ESP distribution of the OTC is similar to that of the TC (Figure 2B). The hydrogen atom in the hydroxyl group is unique to the OTC and has a higher positive ESP.

For CTC molecules, all hydrogen atoms have a high positive ESP. The hydrogen in the hydroxyl group on the D ring has the highest positive ESP (Figure 2C). This may be because the Cl atom, with a high electron-withdrawing ability, is directly connected to the conjugated structure of TCs. Thus, the distribution of π electrons in the CTC is significantly changed.

### 3.2. Molecular Orbital

**Figure 3:**
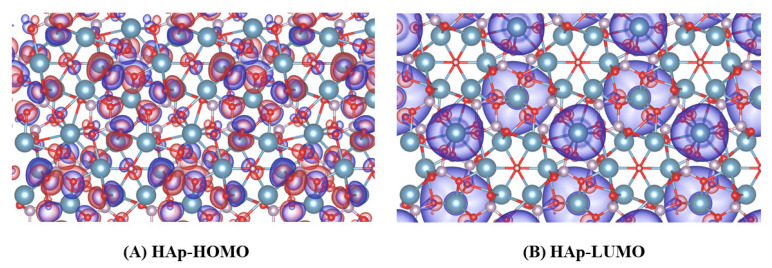
HOMOs and LUMOs of HAp.

**Figure 4:**
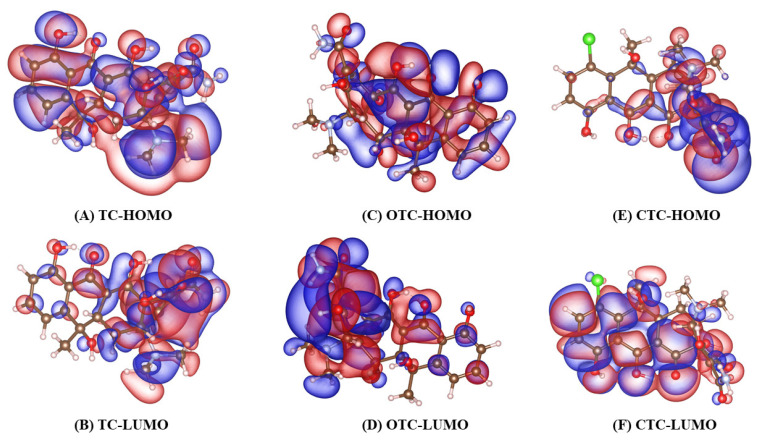
HOMOs and LUMOs of TC, OTC, and CTC.

Figure 3 and Figure 4 show the highest-occupied molecular orbitals (HOMOs) and the lowest-unoccupied molecular orbitals (LUMOs) of HAp and TCs. For HAp, the HOMO is mainly distributed in the phosphate group, whereas the LUMO is mainly distributed on the Ca atom. Similar to the ESP analysis, the Ca atom in HAp is electrophilic (Lewis base), whereas the phosphoric acid group is nucleophilic (Lewis acid).

For the molecular orbitals, the HOMOs of TC and OTC are mainly distributed near the A, B, and C rings, whereas the LUMO is mainly distributed near the D ring. In contrast, for CTC, the HOMO is mainly distributed near the D ring, whereas the LUMO is mainly distributed near the A, B, and C rings.

### 3.3. Adsorption Energy and Geometry

We compared the adsorption energies of the TCs and HAp. The adsorption energy represents the strength of the interaction between the TCs and the surface of HAp.

(1) $$E_{ads}=\left(E_{\mathrm{TCs}}+E_{\mathrm{HAp}}\right)-E_{\mathrm{TCs}+\mathrm{HAp}}$$

where $E_{\mathrm{TCs}+\mathrm{HAp}}$ is the energy of the composite structure after structural optimization, $E_{\mathrm{HAp}}$ is the energy of HAp, and $E_{\mathrm{TCs}}$ is the TC energy.

The adsorption energies are positive; hence, the adsorption between TCs and HAp is thermodynamically favored (Table 1). Moreover, the adsorption energy of OTC is the largest, indicating that OTC has the highest affinity to HAp.

**Figure 5:**
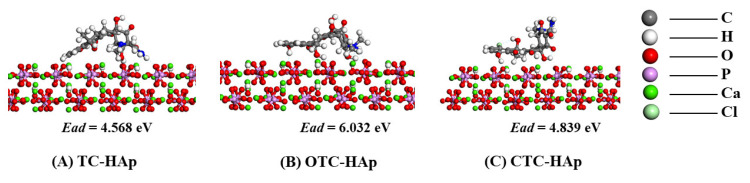
Stable adsorption structures of (**A**) TC, (**B**) OTC, and (**C**) CTC on the HAp surface.

The stable adsorption structure of TCs on the HAp surface after geometric optimization is shown in Figure 5. The adsorption configurations of the three TCs on the HAp surface are significantly different. TC, OTC, and CTC are adsorbed on HAp in an arch shape, a broken-line shape, and a twisted shape, respectively. These adsorption configurations are the same as those of the TCs adsorbed on graphene [43]. The adsorbed configuration may result in a difference in the contact area between them and the HAp surface. This leads to a difference in the adsorption energy. The order of the size of the contact area is OTC-HAp > CTC-HAp > TC-HAp, whereas the order of adsorption energy is OTC-HAp > CTC-HAp > TC-HAp. OTC is adsorbed on HAp in a broken-line shape, possibly because the hydroxyl group introduced on the B ring of OTC changes the hydrogen bond in the OTC molecule, which, in turn, changes the OTC adsorption configuration, exposing more HAp. The binding site increases the adsorption capacity.

### 3.4. IGM Analysis

**Figure 6:**
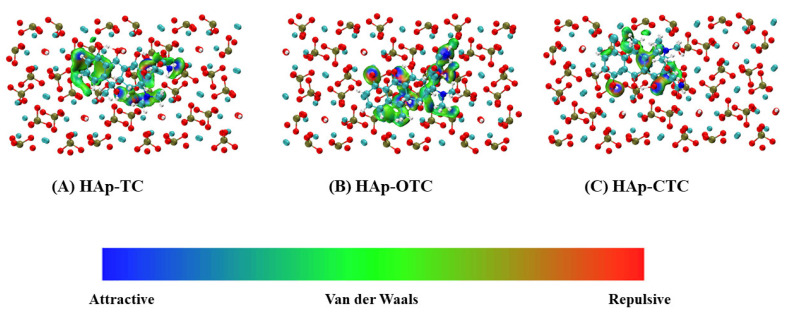
IGM between the two fragments of TCs and HAp.

The IGM method can visualize weak-interaction areas and their characteristics. The calculation depends only on the atomic coordinates; two fragments can be defined as desired [44]. Utilizing the Multiwfn software program [45], we calculated the IGM between the two fragments of TCs and HAp. The calculation results are shown in Figure 6 via Visual Molecular Dynamics (VMD) drawings [46]. 

For TC, the hydrogen atoms on the A ring, in the hydroxyl group on the D ring, and in the amino group produce hydrogen bonds with the oxygen atoms in the phosphate group of HAp. There is a strong van der Waals force between the conjugated structure of the dimethylamino group and the A ring in TC and the HAp surface. 

For OTC, the hydroxyl groups on the A, B, and D rings form strong hydrogen bonds with the phosphate groups on the surface of HAp. The nitrogen atoms in the amino groups on OTC also form strong hydrogen bonds with the hydroxyl groups in HAp. Similarly, the dimethylamino group in OTC can form a weak hydrogen bond with the phosphate group on the surface of HAp.

The interaction between CTC and HAp is weak. Only the hydroxyl group on the A ring forms a hydrogen bond with HAp, whereas the methyl group on the B ring exerts a van der Waals force on HAp. The π electrons in CTC do not interact with the HAp surface, possibly because the electronegative Cl atoms directly connected to the conjugate structure reduce the π electron density.

### 3.5. Analysis of the Charge Density Difference

The formation of coordination bonds leads to violent electron transfer between the two fragments, and this can be clearly observed by performing a charge density difference analysis. The difference in the charge density can be calculated as

(2) $$\Delta \rho =\rho_{\mathrm{HAp}+\mathrm{TCs}}-\rho_{\mathrm{HAp}}-\rho_{\mathrm{TCs}},$$

where $\rho_{\mathrm{HAp}+\mathrm{TCs}}$ represents the charge density of the adsorption complex of HAp and the TCs structure, $\rho_{\mathrm{HAp}}$ represents the charge density of HAp, and $\rho_{\mathrm{TCs}}$ represents the charge density of TCs.

**Figure 7:**
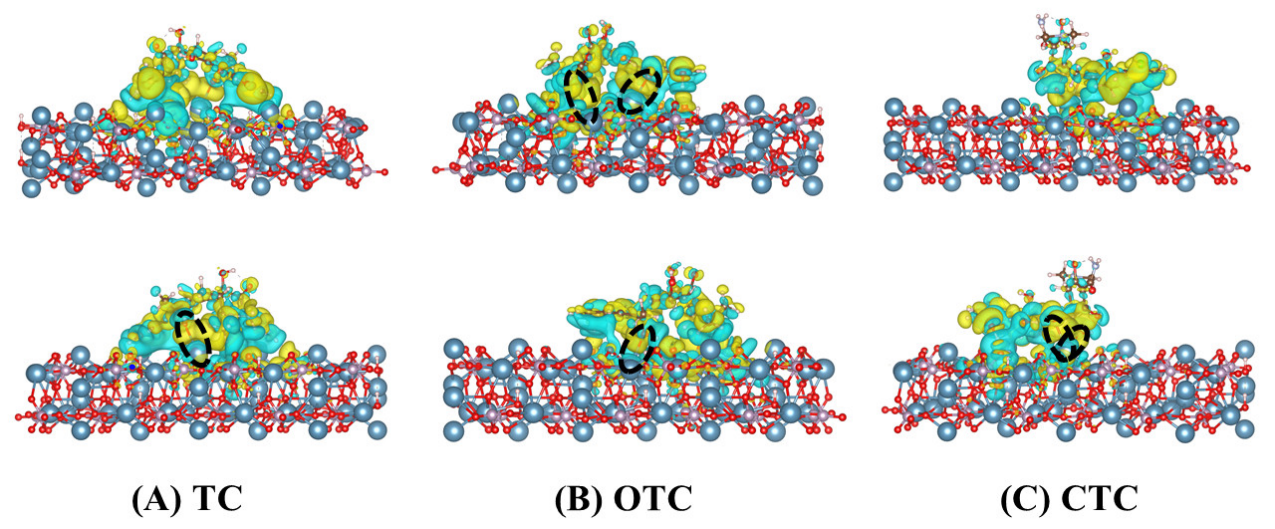
Charge density difference analyses of (**A**) TC, (**B**) OTC, and (**C**) CTC (two side views).

Figure 7 shows the difference in the charge density. The yellow area depicts electron depletion, whereas the cyan area presents electron accumulation. There is a large electron transfer between the oxygen atom in the hydroxyl group on the B ring of TC and the Ca atom on the surface of HAp, thereby forming a coordination bond. Coordination bonds exist between the oxygen atoms in the hydroxyl groups on the B and D rings of OTC and the Ca atoms on the surface of HAp. The carbonyl oxygen on the B ring also forms coordination bonds with the Ca atoms. The two hydroxyl oxygen atoms on the C ring of CTC form a strong coordination bond with the same Ca atom on the surface of HAp.

## 4. Conclusions

In this study, we analyzed the adsorption mechanisms of three TCs and HAp. The conclusions of this study can be summarized as follows.

1. The order of affinity of the three TCs on the surface of HAp was OTC-HAp > CTC-HAp > TC-HAp.
2. In terms of the adsorption configurations of TC-PCL, TC, OTC, and CTC were adsorbed on HAp in an arch shape, a broken-line shape, and a twisted shape, respectively.
3. Both OTC and CTC formed more hydrogen bonds and coordination bonds with HAp. TC exhibited van der Waals interactions and hydrogen bonds but formed only weak coordination bonds.

## Figures and Tables

**Table 1:** Adsorption energy between TC, OTC, CTC, and HAp surfaces (Units: eV).

Models	$E_{TCs+HAp}$	$E_{TCs}$	$E_{HAp}$	$E_{ads}$
TC-HAp	−141,794.223	−7763.023	−134,026.542	4.658
OTC-HAp	−142,227.494	−8194.920	−134,026.542	6.032
CTC-HAp	−142,182.725	−8151.344	−134,026.542	4.839

## Data Availability

Not applicable.
